# Maternal adult attachment and maternal–fetal attachment in the context of romantic relationship quality after premature birth–A cross sectional study

**DOI:** 10.3389/fpsyt.2022.935871

**Published:** 2022-08-23

**Authors:** Karolina Lutkiewicz, Mariola Bidzan

**Affiliations:** Department of Clinical and Health Psychology, Faculty of Social Sciences, Institute of Psychology, University of Gdańsk, Gdańsk, Poland

**Keywords:** attachment style, maternal–fetal attachment, premature birth, relationship quality, depressive symptoms

## Abstract

**Objective:**

The primary outcome of the study was to evaluate the maternal adult attachment and maternal–fetal attachment (MFA) in the context of romantic relationship quality among mothers of preterm born children. Associations between MFA, maternal adult attachment, maternal perceived stress, depressive symptoms, social support in the neonatal period were also examined as secondary outcomes.

**Materials and methods:**

The study had a cross-sectional design and involved 260 of women after premature birth, who participated in the study in the early neonatal period. The following self-reported methods were used: Socio-demographic questionnaire, Attachment Styles Questionnaire (ASQ), Maternal–Fetal Attachment Scale (MFAS), The Perceived Stress Questionnaire (PSQ), The Dyadic Adjustment Scale (DAS), The Edinburgh Postnatal Depression Scale (EPDS), The Social Support Questionnaire (SSQ).

**Results:**

The results showed that a secure attachment style is associated with a higher intensity of the MFA and consequently, a higher quality of the romantic relationship. Regression analysis presented that the higher the secure attachment score, the lower the perceived level of stress and depressive symptoms, which in turn lead to a higher quality of the partner relationship.

**Conclusion:**

Maternal secure attachment positively impacts the romantic relationship quality and the maternal–fetal attachment. The findings also draw attention to the role of the secure attachment style as a protective factor while coping with stress and depressive symptoms.

## Introduction

According to the World Health Organization (WHO) preterm birth occurs when babies are born before the 37th week of pregnancy. The number of premature births has been growing consistently with approximately 15 million children born prematurely worldwide (1). In Poland this rate is between 6.3 and 8% of total births, and in other populations oscillates between 4 and 12% (2, 3). Preterm birth is an important phenomenon due to many potential negative health outcomes and possible long-term complications in the mental and physical development of a child (4). What is more, the experience of preterm birth is considered a stressful and sometimes even traumatic event (5) due to potential negative impacts on child, mother-child dyad, couples’ relationship, and whole family system (6). Therefore, exploring adult attachment style and romantic relationship quality in the context of premature birth is of a great importance.

Based on existing knowledge, relationship quality between parents is an important aspect of a child’s development (7), attachment style (8, 9), language development (10), and the perception of the child (11). Some studies also highlight further outcomes related to the psychological, socio-emotional child development and school performance, which can occur later in life (12) as well as insecure attachment with parents, and worse relationship quality in later life (13, 14). Parental intimacy, constructive communication, warm and empathetic interactions are essential for a child’s development (13–15). What is more, research has shown that parents, who felt supported in their relationship presented more positive parenting behaviors (16). As literature has shown that family dynamics is related with child’s wellbeing, understanding the input of different psychological elements, including adult attachment style, parental mental health as well as exploring the level of contribution of these factors may be a key, especially in the context of prematurity.

One of the significant factors influencing the romantic relationship quality is adult attachment style (17, 18). Adult attachment style is a mental representation of the self in relation with others, which guides individuals in their perceptions, reactions, and expectations in close relationships throughout the lifespan (19). Thus, it is also related with parenthood (18). Adult attachments have been studied in relation to various aspects of parenting. Research suggests that individuals with insecure adult attachment demonstrated fewer behaviors facilitating transition to parenthood than those with a secure attachment style (17). Other studies have shown that maternal adult attachment has an impact on the antenatal attachment with her child (20, 21). Maternal–fetal attachment (MFA) relates to the maternal thoughts, feelings, and behaviors toward her unborn child (22). Brandjerdporn et al. (22) indicated for the link between MFA including different maternal sensory patterns and adult attachment style (22). In another research Chrzan–Detkos and Lockiewicz (20) showed the relationship between maternal adult attachment and antenatal maternal bonding, but not with maternal bonding after birth. In Europe 7–8% of mothers reported a weakened bond with their children in the postnatal period (23). Nordahl et al. (21) found that higher scores on avoidance and anxious attachment styles were related to increased stress, which in turn lead to a decreased quality of maternal bonding (21).

The literature has also presented strong associations between adult attachment styles and mental health. Research showed that adult attachment coexists not only with stress (24), but also between social support (25), depression (26), and anxiety (27). Even though most women perceive parenthood as a positive life event, nevertheless up to 20% of women report anxiety, depression, or stress in the perinatal period (28). As stated above, during pregnancy and the first postnatal year parents are more vulnerable to the experience of stress and various psychological problems, thus, another important aspect that needs to be considered is the psychological wellbeing of parents and its impact on relationship quality.

The connection between mental health and marital satisfaction has been widely studied (29, 30). Research results have reported that a low-quality relationship and lack of satisfaction during the perinatal period was associated with increased risk for depression and anxiety for both men and women (29, 31, 32). In a systematic review of 29 studies, lack of support, especially spousal, was associated with anxiety and depression during pregnancy (33). As presented above, a satisfying romantic relationship significantly impacts an individuals’ general psychological wellbeing, life satisfaction, physical health (34, 35) child development, parental experience, and engagement in the parental role (30). The involvement and performance as a parent can be reduced due to poor relationship quality (36), it has been found that relationship satisfaction predicts emotional availability, infant social-emotional behavior (37), and maternal bonding with a child (38). A systematic review showed that in 9 of 16 studies a higher quality of partner relationship was positively associated with mother-to-infant bonding quality (38).

The child experiences, especially in the first years of their life, are very formative and may have a long-term impact on the child’s development and psychological functioning (14). The parental relationship is found to be a significant component, which determines the quality of family life (7). While a majority of studies concentrate on full term birth, this study focuses on exploring the mother’s attachment style and relationship quality in the context of other psychological variables shortly after the experience of preterm birth.

The literature on the subject presents a broad analysis of factors determining adult attachment style and quality of a partnership relation. However, despite its importance, little is known about the relationship quality among mothers, who experienced preterm birth in relation to maternal attachment style and MFA, including other psychological factors. Research in the early childhood period after preterm birth is limited. The majority of studies researching parental relationship satisfaction are related to adolescence or late childhood. Thus, the present study addresses the early neonatal period, which is a time of forming patterns of interactions and bonding with a baby, through feeding, touching, and spending time cuddling. Neonatal period is a sensitive time for parents who experienced premature birth due to challenges related with preterm birth such as long-term care in a Neonatal Intensive Care Unit, fear for the child’s health, separation of the mother-child dyad, and limitations in holding or touching the baby (5, 39, 40). The experience of prematurity makes these families different and unique in many ways. Preterm birth can be related with more challenges and difficulties than full term birth, which can also impact a partner’s interactions, relationship quality, and parenting. Thus, in this study we decided to focus only on the mothers who experienced preterm birth.

The aim of the study is to obtain more comprehensive picture of psychological factors that are associated with the maternal experience in the post-partum period. The primary goal of this study was to examine the associations between the maternal adult attachment style and MFA, with romantic relationship quality among mothers of premature children. The secondary goal was to examine if there are associations between maternal adult attachment and MFA with maternal perceived stress, depressive symptoms, and social support. Sociodemographic factors were examined with maternal adult attachment and maternal depressive symptoms.

The following research questions were stated:

1. Whether maternal adult attachment is related with the romantic relationship quality among mothers of preterm children?
2. Whether maternal adult attachment is related with depressive symptoms, social support, and perceived stress among mothers of preterm born children?
3. Whether maternal–fetal attachment (MFA) is related with romantic relationship quality and its components?
4. Whether maternal–fetal attachment (MFA) is related with maternal adult attachment, perceived stress, and social support in the neonatal period?
5. Whether maternal adult attachment and depressive symptoms are related with sociodemographic factors (maternal age, maternal education, paternal age and education, marital status, place of living, and financial status)?

## Materials and methods

### Study design and procedures

This cross-sectional study was a part of a broader project conducted in cooperation with the Clinic of Obstetrics at the Medical University of Gdańsk and the University of Gdańsk. The Research Ethics Board at the University of Gdańsk approved the procedures of the study (No. 25/2015).

Enrollment was provided by trained assistants in accordance with predetermined eligibility criteria through medical records of women who gave premature birth–birth to a child born below 37 weeks of pregnancy; preterm birth defined according to the international definition of prematuritym in the Neonatology, Gynaecology and Obstetrics Unit of the University Clinical Center in Gdańsk (Poland). Mothers of newborns after preterm birth were invited to take part in the study by trained assistants after delivery at the University Clinical Center in Gdansk, Poland in accordance with predetermined eligibility criteria through medical records. The study group consisted of mothers who gave birth to a child born below 37 weeks of pregnancy. New mothers were recruited through a personal invitation in the early neonatal period between the first and third day after childbirth–they answered the questionnaires at their convenience between the 1 and 3 day after the birth. Women who agreed to participate in the study, were informed about the procedures of the study and provided written informed consent. It was emphasized that enrollment in the study is voluntary, and parents could refuse to participate without giving any reason. Mothers of prematurely born children were asked to complete paper datasets of questionnaires regarding their subjective assessment of attachment style, relationship quality, perceived stress, depressive symptoms, social support, and MFA. Socio-demographic information were also gathered. Paper datasets were securely stored by the project manager. Each participant received an ID which was retrieved from their medical card. Only mothers in a romantic relationship (formal or informal) with a partner, after preterm birth were considered for inclusion to the study. Exclusion criteria were as follows: a personal decision to not participate in the study, cognitive or physical impairment, or single motherhood.

### Materials and methods

Data in the study was obtained by self-report measures. Demographic characteristics included variables such as: maternal age and education, paternal age and education, place of living, maternal marital status, financial status, number of children in the household, number of pregnancies, gestational age, delivery type, child’s birth weight, and APGAR (Appearance, Pulse, Grimace, Activity, and Respiration) points.

#### Assessment of attachment style

The Attachment Styles Questionnaire (ASQ, (41)) is a tool used to assess adult attachment styles in a romantic relation both directed to men and women (41). This tool was developed by Plopa (41), on the basis of Hazan’s and Shaver’s concept (42). ASQ differentiates three attachment styles: secure, avoidant, and anxious-ambivalent style. The questionnaire consists of 24 items, organized into three scales, related to the above attachment styles. Raw results are calculated into sten scores. The respondents have to answer each of the items by using a seven-point scale. Secure attachment style is related with a high level of security in the relationship, mutual trust, openness in the relationship and a feeling of confidence with a partner in a difficult time. The anxious-ambivalent style is characterized by a lack of security and a high level of anxiety in terms of relationship stability, increased worries, openness, and trust. The avoidant style occurs when a person has difficulty in establishing and maintaining close relationships. The questionnaire has satisfactory methodological properties and criteria. Reliability for the secure style equals 0.91, for the ambivalent-anxious style is 0.78, and for the avoidant style is 0.80 (41). In this study The Cronbach’s alpha reliability coefficient equaled 0.79 for the whole scale.

#### Assessment of relationship quality

The Dyadic Adjustment Scale (DAS, (43); Polish version: (44)) is a 32-item measure designed to assess the relationship quality between married or cohabitating couples. DAS has 32 items, which are organized into four subscales: Dyadic Consensus (the degree to which the couple agrees on matters of importance to the relationship), Dyadic Satisfaction (the degree to which the couple is satisfied with their relationship), Dyadic Cohesion (the degree of closeness and shared activities experienced by the couple), and Affective Expression (the degree of demonstrations of affection and sexual relationships) (43, 44). The results can be calculated by adding the overall score from all the categories. A higher quality of the relationship is indicated by a higher score. Meta-analysis with 128 samples (25,035 participants), reported that the Cronbach’s alpha reliability coefficient for the whole scale was 0.92 and the individual subscales ranged between 0.71 and 0.87 (45). In this study the Cronbach’s alpha reliability coefficient equals 0.925.

#### Assessment of perceived stress

The Perceived Stress Questionnaire (PSQ, (46), Polish version: (41)) is a self-report tool used for the assessment of the general and subjective experience of stress within the last month (41, 46). Originally designed in English, Perceived Stress Questionnaire (PSQ) is a widely used tool, which was validated in many countries and translated into Polish (41), Italian and English (46), Spanish, German, and Swedish (47). The PSQ consists of 30 items, which are organized into seven different components of stress: sense of fatigue (4–16 points), irritability (2–8 points), worries (5–20 points), psychological tension (4–16 points), no joy of life (7–28 points), sense of pressure (4–16 points), and being overwhelmed (4–16 points). The scores are the sum of the results from all the categories. The person can obtain the maximum score of 120, and the minimum is 30 points (41). This instrument can be useful in studying prerequisites and predictors of health and wellbeing in general population (47). The Cronbach’s alpha reliability coefficient equaled > 0.9 (46). In this study the Cronbach’s alpha reliability coefficient equals 0.942.

#### Assessment of depressive symptoms

Depressive symptoms were measured by The Edinburgh Postnatal Depression Scale (EPDS, (48); Polish version: Bielawska-Batorowicz), which is the most common screening tool used worldwide (48). It can be used during pregnancy as well as in the year following the birth of a child. EPDS consists of a set of 10 questions about the symptoms of depression regarding the past 7 days. The scale uses the four-point Likert scale. The total score is calculated by adding the numbers selected for each of the 10 items. The higher the score, the higher the possibility of severe depressive symptoms. The cut-off values of 13/14 or higher are most often used to identify women who might have depression symptoms. The Cronbach’s α coefficients of reliability in a Polish version of EPDS equaled 0.9 (49). The Cronbach’s alpha coefficient in this study calculates as 0.859.

#### Assessment of social support

The Social Support Questionnaire (SSQ, (50); Polish version: (51)), is a quantitative self-report questionnaire designed to measure social support in the postnatal period (50, 51). It contains 14 statements that include three dimensions: social support obtained, desired, and an assessment of satisfaction with support from the subjective perspective of a person. The scale consists of 14 different resources that an individual can receive support from such as family, friends, healthcare workers, church, and community. The items are evaluated based on a four-point scale (1–not at all to 4–extremely agree). The total score ranges between 14 and 56 points (52). The Polish version of the questionnaire for each dimension has the following reliability: desired support (0.85), obtained support (0.69), support assessment (0.88). The Cronbach’s alpha reliability coefficient for the whole scale is 0.864. In this study the Cronbach’s alpha reliability coefficient calculates 0.864.

#### Assessment of a maternal–fetal attachment

Maternal–Fetal Attachment Scale (MFAS, (53); Polish version: (51)) aims to characterize the MFA during pregnancy (51, 53). A 24-item scale is a measure divided into the five following subscales: (a) Taking the parental role, (b) Treating a child as a separate being, (c) Interacting with the child, (d) Assigning characteristics to the child, (e) Being guided by the needs of the child. The questions are evaluated through Likert-type responses. The person answers by selecting one of the following terms: A–definitely yes; B–rather yes; C–I find it difficult to answer; D–probably not; E–definitely not. The final score is obtained by the sum of all responses, which vary from 24 to 120 points. The higher the score obtained by the respondent, the higher MFA. Reliability for the Polish version for the entire questionnaire is calculated as 0.81, and for the scales, respectively: 0.66; 0.58; 0.52; 0.61; 0.50 (51). In this study the Cronbach’s alpha reliability coefficient for the whole scale is 0.726.

### Data analysis

Data analysis was obtained using the Statistical Package for Social Science (SPSS) version 24. In the first step of the statistical analysis, descriptive statistics were calculated. In the second step, initial associations between measured variables were obtained through Persons’ correlations. In the third step, linear hierarchical regression was used to examine whether attachment style is a predictor of relationship quality and finally individual pathways of the studied mediation models were calculated. Then, bootstrap testing for indirect effects was performed. To analyze the internal consistency of all research tools, reliability measures were calculated using the Cronbach’s alpha method. On the basis of the conducted analyses, it can be concluded that all research tools have reliability above 0.7, which means their internal consistency is high.

## Results

### The characteristics of the study group

A total of 260 women participated in this cross-sectional study who gave birth to a premature child. The majority of mothers (161 mothers, 79.7%). 60.3% (129 mothers) of respondents had a university degree, 75.9% (129 mothers) of participants reported living in a city with a population above 100,000 or more. The majority of women 75.4% (196 mothers) were pregnant with one child–single pregnancy as well as most of the women were primiparas (63.3%, 164 mothers). What is important, 66.3% (126 mothers) of women were hospitalized during their pregnancy. In 9 out of 10 cases (90.0%) the child was put into an incubator after birth. To conclude, most children were late-term preemies (78.4%, 200 infants). 14.5% of children (37 children) were born as mid-term preemies and 7.1% as early preterm infants (18 infants). Socio-demographic factors were not analyzed in this article with all psychological variables, as some of them were presented in the separate article (54). Socio-demographic variables of the study sample are presented in Table 1.

**TABLE 1:** Socio-demographic variables of the study sample.

Variable	*M* (*SD*)/Med	%	Minimum	Maximum
Maternal age	30.00 (5.22)	–	18.0	45.0
Maternal education	–	60.3% post-secondary	–	–
Paternal age	32.07 (5.78)	–	17.0	51.0
Paternal education	–	48.3% higher education	–	–
Place of living	–	75.9% city	–	–
Number of children	–	62.8% no children	0	4
Financial status	–	49.6% good	–	–
Gestational age	34.0	–	24	36
APGAR (infant)	8.18 (1.18)	–	3	10
Birth weight (infant)	2229.40 (731.79)	–	580	4160
Delivery type	–	36.7% natural birth	–	–

### Maternal adult attachment and the romantic relationship quality among mothers

To answer the first research question whether maternal adult attachment is related with the relationship quality among mothers of preterm children, correlation analysis was conducted. Correlation analysis revealed that secure attachment style correlated positively with all dimensions of relationship quality. All correlations were positive and strong. The strongest one occurred between the secure attachment style and the general relationship quality (*r* = 0.72, *p* = 0.01). Maternal anxious attachment style correlated negatively with all dimensions of the relationship quality, apart from cohesion. All correlations observed were moderately strong. No statistically significant correlation was observed between the quality of the relationship quality and the avoidant attachment style (Table 2).

**TABLE 2:** Pearson’s linear correlation coefficient for the relation between general satisfaction with the relationship quality and its components with maternal adult attachment.

Variable	*M* (*SD*)	Secure attachment	Anxious attachment	Avoidant attachment
Consensus	52.78 (7.24)	0.69**	–0.34**	–0.05
Cohesion	19.53 (3.68)	0.50**	–0.06	0.07
Satisfaction	41.32 (5.25)	0.643*	–0.35**	–0.08
Emotional expression	10.42 (1.74)	0.44**	–0.32**	–0.07
General satisfaction	124.05 (15.09)	0.72**	–0.33**	–0.04

*M*, mean; *SD*, standard deviation.

*p < 0.05; **p < 0.01.

Source: own work.

### Maternal adult attachment and its association with depressive symptoms, social support, and perceived stress

To answer the second research question whether maternal adult attachment is related with depressive symptoms, social support, and perceived stress among mothers of preterm born children correlation analysis was conducted. Correlation analyses showed that the anxious attachment style positively correlated with depressive symptoms (*r* = 0.43, *p* = 0.01). The correlation between the anxious attachment style and perceived stress was very strong (*r* = 0.90, *p* = 0.01). Similar associations were obtained regarding the avoidance attachment style, but the strength of these correlations was weaker. The results also show that the secure attachment style negatively correlated with maternal depressive symptoms and perceived stress. In terms of attachment style and social support dimensions, there was only one significant correlation (weak and negative) between anxious attachment style and the quality of the received support (*r* = -0.15, *p* = 0.05). All results are presented in Table 3.

**TABLE 3:** Pearson’s linear correlation coefficient for the relationship between maternal adult attachment styles with depression, social support, and perceived stress.

Variable	*M* (*SD*)	Anxious attachment	Avoidant attachment	Depressive symptoms	Desired social support	Received social support	Quality of received support	Perceived stress
Secure attachment	3.74 (0.41)	–0.24**	0.01	–0.27**	0.05	0.08	0.09	0.24**
Anxious attachment	1.93 (0.52)		0.39**	0.43**	0.04	–0.10	–0.15*	0.90**
Avoidant attachment	2.04 (0.46)			0.21*	0.09	–0.004	–0.09	0.43**
Depressive symptoms	10.14 (5.55)				0.01	–0.06	–0.12	0.41**
Desired social support	37.61 (7.04)					0.61**	0.49**	0.06
Received social support	33.62 (5.41)						0.87**	–0.09
Quality of received support	34.26 (6.01)							–0.10
Perceived stress	56.47 (17.1)							

*M*, mean; *SD*, standard deviation.

*p < 0.05; **p < 0.01.

Source: own work.

Table 4 presents detailed analysis of correlations between adult attachment and perceived stress components. The anxious and avoidant attachment styles correlated positively with the sense of fatigue, irritability, worries, psychological tension, lack of joy of life, sense of pressure, and being overwhelmed. All correlations between the anxious style and all stress dimensions were very strong (above *r* = 0.70, *p* = 0.01). Meanwhile, correlations between the maternal avoidant attachment and all stress dimensions were statistically significant, but moderately strong. The maternal secure attachment style correlated negatively with the anxious attachment style, irritability, worries, psychological tension, lack of joy of life sense of pressure, and being overwhelmed. All the correlations were weak (Table 4).

**TABLE 4:** Pearson’s linear correlation coefficient for the relation between maternal adult attachment and the perceived stress components.

Variable	*M* (*SD*)	Anxious	Avoidant	Sense of fatigue	Irritability	Worries	Psychological tension	Lack of joy in life	Sense of pressure	Being overwhelmed
Safe attachment	3.74 (0.41)	–0.24**	0.01	–0.10	–0.25**	0.20**	–0.21**	0.28**	–0.18**	–0.18**
Anxious attachment	1.93 (0.52)		0.39**	0.75**	0.70**	0.79**	0.74**	0.74**	0.74**	0.80**
Avoidant attachment	2.04 (0.46)			0.35**	0.32**	0.40**	0.38**	0.37**	0.31**	0.38**

*M*, mean; *SD*, standard deviation.

*p < 0.05; **p < 0.01.

Source: own work.

### Maternal–fetal attachment and romantic relationship quality and its components

To answer the third research question whether MFA is associated with romantic relationship quality and its components, correlation analysis was conducted. According to the stated hypothesis, the MFA is related to the romantic relationship quality in a group of mothers. The analysis showed that the MFA is positively correlated with the romantic relationship quality and all its dimensions in the neonatal period in mothers of premature babies (Table 5).

**TABLE 5:** Pearson’s linear correlation coefficient for the relation between relationship quality and maternal–fetal attachment (MFA).

Variable	*M* (*SD*)	MFA
Consensus	52.78 (7.24)	0.24**
Cohesion	19.53 (3.68)	0.15*
Satisfaction	41.32 (5.25)	0.13*
Emotional expression	10.42 (1.74)	0.18**
General satisfaction	124.05 (15.09)	0.22**

*M*, mean; *SD*, standard deviation.

*p < 0.05; **p < 0.01.

Source: own work.

### Maternal–fetal attachment and the association with maternal adult attachment, perceived stress, and social support in the neonatal period

To answer the fourth research question whether MFA is associated with maternal adult attachment, perceived stress, and social support in the neonatal period correlation analyzes was conducted. It was examined whether MFA coexists with other psychological factors (maternal stress, social support and maternal adult attachment style). It was shown that MFA during pregnancy positively correlated with the obtained social support and the quality of the obtained social support. What is more, MFA coexisted with the maternal secure attachment style, however, there were no associations between anxious and avoidant attachment styles and MFA (Table 6).

**TABLE 6:** Pearson’s linear correlation coefficient for the relation between maternal–fetal attachment (MFA), social support, and perceived stress and maternal adult attachment.

Variable	*M* (*SD*)	Perceived stress	Perceived social support	Received social support	Quality of social support	Secure attachment	Anxious attachment	Avoidant attachment
MFA	99.41 (9.61)	–0.13	0.12	0.14*	0.14*	0.16*	–0.10	–0.02

*M*, mean; *SD*, standard deviation.

*p < 0.05; **p < 0.01.

Source: own work.

Detailed correlation analysis related to stress and its component showed that MFA correlated negatively with a sense of fatigue, irritability, psychological tension, and lack of joy in life (Table 7).

**TABLE 7:** Pearson’s linear correlation coefficient for maternal–fetal attachment (MFA) and maternal perceived stress with its components.

Variable	*M* (*SD*)	Perceived stress	Sense of fatigue	Irritability	Worries	Psychological tension	Lack of joy in life	Sense of pressure	Being overwhelmed
MFA	99.41 (9.61)	–0.13	–0.15*	–0.17*	–0.08	–0.16*	0.19**	–0.09	–0.11

*M*, mean; *SD*, standard deviation.

*p < 0.05; **p < 0.01.

Source: own work.

### Sociodemographic factors and their associations with maternal adult attachment and depressive symptoms

To answer the question whether maternal adult attachment and depressive symptoms are related with sociodemographic factors (maternal age, maternal education, paternal age and education, marital status, place of living, and financial status) correlation analysis was conducted. Analyzes of the associations between the attachment style and social factors showed that only the anxious attachment style negatively correlated with the maternal education, paternal education, and the mother’s material status. Depressive symptoms negatively correlated with the paternal education, maternal marital status, and mother’s place of residence. Moreover, the correlation between marital status and maternal depressive symptoms is statistically significant. On this basis, it can be concluded that the informal partnership coexists with a risk of post-partum depression in mothers of premature babies. The collective results are presented in Table 8.

**TABLE 8:** Pearson’s linear correlation coefficients and point-two-series correlation coefficients for the relationship of maternal adult attachment and sociodemographic variables.

Variable	*M* (*SD*)	Maternal age*^a^*	Paternal age*^a^*	Maternal education*^b^*	Paternal education*^b^*	Marital status*^b^*	Place of living*^b^*	Financial status*^b^*
Secure attachment	3.74 (0.41)	–0.13	–0.07	0.06	0.07	0.05	0.24**	0.02
Anxious attachment	1.93 (0.52)	0.02	–0.04	–0.21**	–0.25**	–0.06	–0.10	–0.18**
Avoidant attachment	2.04 (0.46)	0.07	–0.04	–0.07	–0.13	–0.004	0.07	–0.03
Depressive symptoms	10.14 (5.55)	0.07	0.04	–0.15	–0.22**	–0.23**	–0.22**	–0.05

Annotation: Maternal and paternal education, Marital status (0–informal relationship, 1–married), (0–lower than average or average, 1–good or very good).

*^a^*Pearson’s linear correlation coefficients.

*^b^*Point-two series correlation coefficients.

*p < 0.05; **p < 0.01.

Source: own work.

### Regression analysis

A further hierarchical regression analysis was conducted to examine if the maternal adult attachment is a predictor of the romantic relationship quality. The results showed that secure attachment style is significantly associated with romantic relationship quality. Secure attachment style is related to relationship quality and all relationship quality components (consensus, cohesion, emotional expression, satisfaction). Obtained results are presented in the Tables 9A,B. Based on the regression analysis in the conducted study, the strongest predictor of the romantic relationship quality is the maternal secure attachment style in her relationship with the partner. The results are presented in Tables 9A,B.

**TABLE 9A:** Hierarchical regression analysis, where consensus, cohesion and satisfaction were explained variables.

	Consensus		Cohesion		Satisfaction	
			
Step predictor	β	Δ *R*^2^	*B*	Δ *R*^2^	β	Δ *R*^2^
Secure attachment style	0.57**		0.44**		0.55**	
Avoidant attachment style	0.07		0.09		0.05	
*R* ^2^		0.563**		0.312**		0.487**

β, the standardized beta; B, the unstandardized beta; Δ*R*^2^, the coefficient of determination. *p < 0.05; **p < 0.01.

Source: own work.

**TABLE 9B:** Hierarchical regression analysis, where emotional expression and general satisfaction were explained variables.

	Emotional expression		General satisfaction	
		
Step predictor	β	Δ *R*^2^	*B*	Δ *R*^2^
Secure attachment style	0.36**		0.61**	
Avoidant attachment style	0.08		0.08	
*R* ^2^		0.336**		0.595**

β, the standardized beta; B, the unstandardized beta; Δ*R*^2^, the coefficient of determination. *p < 0.05; **p < 0.01.

Source: own work.

Regression analyzes were performed to investigate the individual pathways of the studied mediation models. In the first model, the results first showed a significant association between the adult secure attachment and general satisfaction with the romantic relationship (path c). Secondly, the results showed a significant association between the secure attachment style and perceived stress (path a). Third, the results showed a significant association between perceived stress and general satisfaction with the relationship (path b) (see Table 10). Then, bootstrap testing for indirect effects was performed. The results showed that perceived stress was a partial mediator of the association between the secure attachment style and general satisfaction with the romantic relationship. The indirect effect was statistically significant and amounted to *B* = 2.32. The direct effect of the secure attachment style on the overall satisfaction with the relationship was statistically significant and amounted to *B* = 24.82.

**TABLE 10:** Non-standardized regression coefficients, Student’s *t*-test values, and *p*-value for individual pathways in mediation models.

			Path	*B*	*t*	*p*
Maternal–fetal attachment	←	Secure attachment style	A	3.83	2.34	0.021
Relationship quality	←	Maternal–fetal attachment	B	0.22	2.80	0.006
Relationship quality	←	Secure attachment style	C	27.14	14.72	<0.001
Relationship quality	←	Secure attachment style	c’	26.31	14.32	<0.001
Perceived Stress	←	Secure attachment style	A	–8.86	–3.86	<0.001
Relationship quality	←	Perceived Stress	B	–0.26	–4.92	<0.001
Relationship quality	←	Secure attachment style	C	27.14	14.72	<0.001
Relationship quality	←	Secure attachment style	c’	24.82	13.72	<0.001
Depressive symptoms	←	Secure attachment style	A	–3.48	–2.90	0.005
Relationship quality	←	Depressive symptoms	B	–0.41	–2.34	0.021
Relationship quality	←	Secure attachment style	C	29.19	13.09	<0.001
Relationship quality	←	Secure attachment style	c’	27.78	12.25	<0.001

In the second model, first the results showed a significant association between the secure attachment style and overall satisfaction with the relationship (path c). Second, the results showed a significant association between the secure attachment style and depressive symptoms (path a). Third, the results showed a significant association between depressive symptoms and overall satisfaction with the relationship (lane b) (see Table 10). Then, bootstrap testing for indirect effects was performed. The results showed that depressive symptoms was a partial mediator of the association between the secure attachment style and overall satisfaction with the relationship. The indirect effect was statistically significant and amounted to *B* = 1.41. The direct effect of the secure attachment style on the general satisfaction with the relationship was statistically significant and amounted to *B* = 27.78.

In the third model, first, the results showed a significant association between the secure attachment style and general satisfaction with the relationship (path c). Second, the results showed a significant association between the secure attachment style and MFA (path a). Third, the results showed a significant association between MFA and general satisfaction with the relationship (path b) (see Table 10). Then, bootstrap testing of indirect effects was performed. The results showed that MFA was a partial mediator of the association between the secure attachment style and overall satisfaction with the relationship. The indirect effect was statistically significant and amounted to *B* = 0.83. The direct effect of the secure attachment style on the overall satisfaction with the romantic relationship was statistically significant and amounted to *B* = 26.31.

Table 11 presents the values of direct and indirect effects as well as the value of the *R*^2^ coefficient together with 95% confidence intervals in individual mediation analyzes.

**TABLE 11:** Values of direct and indirect effects as well as the value of the *R*^2^ coefficient together with 95% confidence intervals in individual mediation analyzes.

Independent variable	Dependent variable	Direct effect	*95% CI*	Indirect effect	*95% CI*	*R* ^2^	*95% CI*
Perceived stress	Relationship quality	–0.08	–0.21 to 0.009	–0.30	–0.47 to –0.13	0.07	0.02 to 0.16
Depressive symptoms	Relationship quality	–0.37	–0.82 to –0.12	–0.56	–1.09 to –0.03	0.07	0.02 to 0.16
Secure attachment style	Relationship quality	0.83	0.16 to 2.25	26.31	22.69 to 29.94	0.04	0.005 to 0.10
Secure attachment style	Relationship quality	2.32	1.14 to 3.94	24.82	21.26 to 28.39	0.11	0.05 to 0.19
Secure attachment style	Relationship quality	1.41	0.17 to 3.61	27.78	23.28 to 32.27	0.10	0.01 to 0.23

## Discussion

### Maternal adult attachment and romantic relationship quality of mothers of preterm children

Firstly, in this study, it was found that a maternal secure attachment style to her partner is positively related with the romantic relationship quality and its’ all dimensions. However, regression analysis showed that only the secure attachment style was the predictor of the relationship quality among mothers of preterm born children. These findings suggest that the adult secure attachment style fosters and increases the quality and satisfaction with the relationship quality and is connected with all components of the relationship quality: consensus, cohesion, emotional expression, and satisfaction with the relationship. A similar result was obtained by (55), where attachment security was one of the main predictors of romantic relationship quality (55). What is more, among first time parents, securely attached individuals reported higher relationship quality (56), and a higher satisfaction from the relationship than those with other attachment styles (42, 57, 58). Such results can be due to the fact that individuals with the secure attachment style have a positive image of themselves and the world, are featured by a greater trust and self-confidence, and are more open to their partners (59). In contrast, individuals with the anxious attachment style often have a negative self-image and describe themselves as unable to trust another person, experiencing a constant and intense fear of rejection. Relationships based on the anxious-ambivalent and avoidant attachment styles are featured by a higher rate of conflicting behaviors, a lower level of intimacy, involvement, and satisfaction (58). Obtained results in this study showed that anxious attachment style is related negatively with all dimensions of relationship quality, apart from cohesion. What is interesting, this research has found no significant correlation between the avoidant attachment style and the romantic relationship quality or its dimensions. Knoke et al. (59) have found that only the anxious attachment style is a significant indicator of the partnership relation’s quality that leads to a decrease in this quality (59). However, meta-analyses reported that both insecure attachment styles, anxious and avoidant, are associated with dissatisfaction in the relationship and a lower quality of the relationship (60, 61). Insecure attachment styles in this study were not predictors of romantic relationship satisfaction. As presented above, these results are partially in line with the other research, where attachment styles are important predictors of the partnership relation’s quality and satisfaction (18, 59, 62). It must be mentioned that the mechanisms of attachment styles are not yet fully understood and there are differences in research results. More information is needed to better understand the specific strategies and coping of individuals with higher levels of insecure attachments in specific situations (25) such as preterm birth.

### Maternal adult attachment and its association with maternal depressive symptoms, social support, and perceived stress

In the second step, we examined whether maternal adult attachment is related to depressive symptoms, social support, and perceived stress among mothers of preterm born children. The conducted research has revealed that the avoidant and anxious attachment style is associated with a higher sense of stress and higher scores in depressive symptoms. Many research projects have been focused on investigating the relation between an individual attachment style and the risk of developing depression or a sense of stress (63, 64). Some studies have showed that anxious and avoidant attachment styles are related to higher levels of parental stress (21, 65). The results of the authors’ own research indicate that the anxious and avoidant attachment style of mothers of preterm children is related to their higher irritability, worrying, psychological tension, lack of joy of life, sense of pressure, and being overwhelmed. It can be said that avoidant or anxious attachment styles make partners more susceptible to the influence of distress (66). Furthermore, in this study it was found that the insecure attachment styles pose a risk of post-partum depressive symptoms in mothers of preterm babies. This result corresponds with those obtained in a systematic review, where eight studies found both anxious and avoidant styles to be related with maternal postnatal depression (26). Based on the obtained results, it can be claimed that a maternal secure attachment style is a protective factor in relation to depressive symptoms, perceived stress and their derivatives. Hirscherberger et al. (56) also suggested that the secure attachment style prevents the development of a high level of stress in a relationship, as compared to other attachment styles (56). However, it must be highlighted that data explaining the coexistence of these factors in the transition to parenthood period in the case of mothers of preterm children, are still missing. In further mediation analysis, perceived stress and depressive symptoms experienced by mothers of prematurely born children were found to be mediators between secure attachment style and relationship quality in mothers. It can be said that a secure attachment style is associated with a lower level of perceived stress and depressive symptoms, which leads to a higher quality of the partner relationship. To our knowledge, no previous studies have directly assessed the relationships between adult attachment style, stress, depressive symptoms, and the quality of relationships between parents.

To conclude, it is very important to look closer at the role of adult attachment styles on the perception of social stressors and difficult emotions. People with insecure attachment styles demonstrate negative mental processing and vulnerability to stressful events, and they are more reactive toward such situations. Individuals with a secure attachment style have a more positive inner faith toward social support in stressful situations (67). Research has shown that securely attached individuals report higher levels of perceived support and higher satisfaction with received support in comparison with insecurely attached people (68). In this study there was only one significant correlation between anxious attachment style and the quality of the received support. The result is rather surprising, as according to the Bartholomew and Horowitz’s (69) model of attachment patterns, people with secure attachment are more willing to look for support, as in general they build more supportive and fulfilling relationships, whereas people with insecure attachment styles are more likely to perceive others’ responsiveness in a negative way (18, 70).

### Maternal–fetal attachment and romantic relationship quality among mothers

In the second part of this study, MFA with the romantic relationship quality and its components was examined. It was found that MFA positively correlated with the quality of the romantic relationship and all its dimensions in the neonatal period. Research conducted by Bieleninik et al. (70) also showed that there is an association between the intensity of the emotional bond between the mother and the child and the high quality of the relationship, especially in multiple pregnancies (70). Other researchers have shown that a positive relationship between partners is one of the determinants of a strong emotional attachment with the child (71, 72).

### Maternal–fetal attachment and the associations with maternal adult attachment, perceived stress, and social support

The research of attachment styles indicates the important role of the maternal adult attachment in the process of transition to parenthood and shaping the relationship and bond with the newborn child (20, 73, 74, 75). Thus, in further step of the analysis, it was examined whether MFA coexists with maternal adult attachment. Studies reported that mothers with secure attachment styles have more positive mother–infant bonding during the first year post-partum (76, 77), while mothers attached insecurely reported some disruptions to the mother-infant bond (78). Mikulincer and Florian (1999) found that women with secure romantic attachments develop stronger bonds with their children during the first trimester of pregnancy compared to anxious-ambivalent and avoidant women (79). In this study we found that MFA coexisted with the mother’s adult secure attachment style. This can be explained that securely attached women to their partners activate their attachment style while transitioning to parenthood, which impacts the imaginary connection with the fetus. Thus, they are more prone to grow a positive prenatal bond with unborn child (80). What is more, mediation analysis showed that MFA is a mediator between the adult secure attachment and the quality of the relationship. The results suggest that the maternal adult secure attachment style is associated with a higher intensity of MFA and, consequently, a higher quality of the relationship. To our knowledge, there are no studies examining the associations between maternal adult attachment style, MFA and romantic relationship quality. Surprisingly in this study there were no associations between maternal anxious and avoidant attachment style with their partner and MFA. In contrary to those results, Ponti et al. (80) found that both insecure attachment styles were significantly connected with low levels of prenatal attachment between a mother and a baby (80). Mothers who claimed anxious and avoidant attachments with their partners also reported a lower proportion of positive and warm feelings toward their unborn baby (80).

In the final step of the analysis, the associations between MFA with the perceived stress and social support were examined. Correlation analysis showed that they were negative correlations between MFA and perceived stress and its components such as the sense of fatigue, irritability, psychological tension, and lack of joy in life. Adapting to motherhood is a potentially stressful process, which activate many positive and negative reactions and feelings (81). Complications in the course of pregnancy and/or during labor, negative reproductive experiences, high-risk pregnancy may cause additional stress and a high level of anxiety among new mothers. High psychological distress, a composite of depressive, anxious, and stress-related symptoms, was found to be associated with lower maternal fetal bond (82). Nordahl et al. (21) found that each attachment style (analyzed separately) and maternal infant bonding was mediated by experienced stress, which suggest that stress is an important factor associated with the maternal bond with a child (21, 83).

A large body of literature points that social support, types of support, its impact on distress and access to the supportive resources for women after childbirth are vital for positive outcomes for women after they have had a baby (84). In this study it was also showed that the obtained social support and the quality of the obtained social support had a positive correlation with MFA. This result is in line with results of systematic review conducted by McNamara et al. (72), where six out of seven studies found that higher MFA was connected with the higher level of social support (72). In other studies, received social support by the mother was among the determinants of the mother-child bond (85, 86, 75). Thus, it can be claimed that having supportive relationships with significant others can foster a better emotional connection with a child (80).

### Strengths and limitations

The definite strength of this study is its conduction in the early neonatal period and including mothers of preterm born children. The presented study is novel, as it demonstrates further associations between maternal adult attachment, MFA and relationship quality, including other important psychological factors such stress, depressive symptoms and social support, which previously have not been fully explored in the research of the subject. Those psychological factors often play an important role in the post-partum period, so we obtained a more comprehensive picture of the factors that are associated with the maternal experience in the post-partum period.

On the other hand, there are several limitations in this study, which deserve attention. The main limitation of the study is the impossibility of making a comparison of psychological conditions of the partnership relation’s quality in the group of mothers with different experience of prematurity based on gestational age. Secondly, in this study most women gave birth to late-term preemies (78.4%), and data including group of infants classified as early premature children (between the 24th and 33rd week) was insufficient to conduct satisfying statistical analyses. However, it has to be mentioned that the distribution of preemies (divided into early, mid-term, or late-term) presented in the research is congruent with the distribution of preemies in the general population. Furthermore, the size of the sample group is rather small, which hinders the inclusion of more variables in the regression analysis, thus limiting the possibilities for testing different, more complex models of associations among the variables. Another definite limitation is a lack of comparison group of mothers after full term delivery and their relationship experiences. The lack of a comparison with full-term infants, and the lack of variation within the preterm birth group, makes it difficult to understand whether obtained findings are a function of the general factors or instead something specific to parents of premature infants. All the measures were self-report questionnaires, so the data is based solely on maternal caregiver report, thus, the interpretation of the conclusion should be done with caution. Also, it has to be mentioned that sociodemographic variables were not included in the analysis, as they were discussed in the previous article (54).

Future studies should be conducted using a longitudinal design. Examining the changes in the relationship quality, monitoring its associations with the maternal bond and parental mental health during pregnancy and the postnatal period would be beneficial. Also, it would be interesting to find how exactly secure attachment style plays a role as a protective factor after preterm birth including in the study design control group of non-premature birth. Exploration of attachment styles high in anxiety and avoidance is also needed, as it is unknown how exactly they impact maternal–fetal attachment after the premature birth.

## Conclusion and clinical implications

The paper enhances further understanding of maternal adult attachment, MFA, and its connections with relationship quality, stress, depressive symptoms, and social support. It can be concluded that adult secure attachment style can be a protective factor while experiencing stressful situations or coping with depressive symptoms. Also, it positively impacts the romantic relationship quality and maternal–attachment. These findings draw attention to the importance of adult attachment and suggest that secure attachment may promote positive relational outcomes for parents, couples, and families during the transition to parenthood. Postnatal screening of attachment, maternal–infant bond, maternal mental health, and relationship quality style should be implemented, especially after preterm birth, in order to detect potential difficulties that mothers and their families can experience. Interventions among mothers should be tailored based on the various attachment styles as the needs and ways of coping with difficulties during pregnancy and postnatal period may be different.

The results pay attention also to the earlier assessment of maternal psychological wellbeing during pregnancy, including an assessment of maternal adult attachment, which may help to identify high risk mothers and initiating interventions to prevent adverse neonatal and developmental outcomes. During perinatal and postnatal care, home visiting programs may allow early detections of challenges and problems that mothers experience and help while working with maternal adult attachment style to build secure mother–baby attachment beginning in pregnancy. The obtained results can be applied in individual and family therapies to target interpersonal conflicts contributing to mood and anxiety disorders. Individual and couples’ counseling should also be available in the perinatal and post-partum period as a supportive resource for parents as working toward a satisfactory relationship would enhance and support the process of bonding with a child.

## Data availability statement

The raw data supporting the conclusions of this article will be made available by the authors, without undue reservation.

## Ethics statement

The study protocol was approved by the Research Ethics Board at the University of Gdańsk, Poland (number 25/2015). The patients/participants provided their written informed consent to participate in this study.

## Author contributions

KL conceived the study, analyzed the data, and wrote the manuscript. KL carried out data collection under the supervision of MB. Both authors designed the study, critically revised the manuscript for publication, and approved the final version.

## Abbreviations

MFA: maternal–fetal attachment
ASQ: Attachment Styles Questionnaire
PSQ: The Perceived Stress Questionnaire
DAS: The Dyadic Adjustment Scale
EPDS: The Edinburgh Postnatal Depression Scale
SSQ: The Social Support Questionnaire
MFAS: Maternal–Fetal Attachment Scale
WHO: World Health Organization
APGAR: Appearance, Pulse, Grimace, Activity, and Respiration.
